# Neuromuscular Junction Dismantling in Amyotrophic Lateral Sclerosis

**DOI:** 10.3390/ijms18102092

**Published:** 2017-10-03

**Authors:** Valentina Cappello, Maura Francolini

**Affiliations:** 1 Center for Nanotechnology Innovation@NEST, Istituto Italiano di Tecnologia Piazza San Silvestro 12, 56127 Pisa, Italy; 2 Department of Medical Biotechnology and Translational Medicine, Università degli Studi di Milano—Via Vanvitelli 32, 20129 Milano, Italy

**Keywords:** neuromuscular junction, Amyotrophic Lateral Sclerosis, tripartite synapse

## Abstract

Neuromuscular junction assembly and plasticity during embryonic, postnatal, and adult life are tightly regulated by the continuous cross-talk among motor nerve endings, muscle fibers, and glial cells. Altered communications among these components is thought to be responsible for the physiological age-related changes at this synapse and possibly for its destruction in pathological states. Neuromuscular junction dismantling plays a crucial role in the onset of Amyotrophic Lateral Sclerosis (ALS). ALS is characterized by the degeneration and death of motor neurons leading to skeletal muscle denervation, atrophy and, most often, death of the patient within five years from diagnosis. ALS is a non-cell autonomous disease as, besides motor neuron degeneration, glial cells, and possibly muscle fibers, play a role in its onset and progression. Here, we will review the recent literature regarding the mechanisms leading to neuromuscular junction disassembly and muscle denervation focusing on the role of the three players of this peripheral tripartite synapse.

## 1. The Neuromuscular Junction as a Tripartite Synapse

At the end of the 1990, it was proposed that glial cells, besides providing the ideal milieu for neuronal function, might have a role in the modulation of neuronal activity, synaptic neurotransmission, and plasticity. It was shown that they were able to respond to neuronal activity by increasing their intracellular Ca^2+^ concentration [1] and releasing chemical gliotransmitters (i.e., glutamate, d-serine, and adenosine triphosphate (ATP) from astrocytes) [1,2,3], thus modulating the activity of neurons and the strength of their signaling. These considerations led to the definition of the tripartite synapse, where not only the pre- and postsynaptic neurons, but also glial cells, were active partners. Even if the concept of tripartite synapse was initially developed for glutamatergic synapses in the central nervous system (CNS), early studies demonstrated that terminal Schwann cells (TSCs) (or perisynaptic Schwann cells) were equally able to modulate acetylcholine release at the frog neuromuscular junction (NMJ) [3] and, to date, the NMJ is considered a peripheral tripartite synapse that is formed by the motor neuron nerve ending, the postsynaptic membrane on skeletal muscle fiber and the TSCs.

The role of these three cellular components in the physiology of the NMJ has been extensively investigated; nonetheless, the contribution of TSCs and muscle fibers in NMJ disassembly in pathological states is still a matter of debate. The scope of this review is to summarize the recent literature describing the contribution of all the elements of the NMJ in the events leading to skeletal muscle denervation in Amyotrophic Lateral Sclerosis (ALS). For a better understanding of these pathological events it is necessary to recapitulate which are the main steps in the assembly and maintenance of the NMJ as, as it is often the case, these might be altered in degenerative processes.

## 2. Neuromuscular Junction Assembly and Plasticity

### Cross-Talk during Neuromuscular Junction Assembly

**Figure 1:**
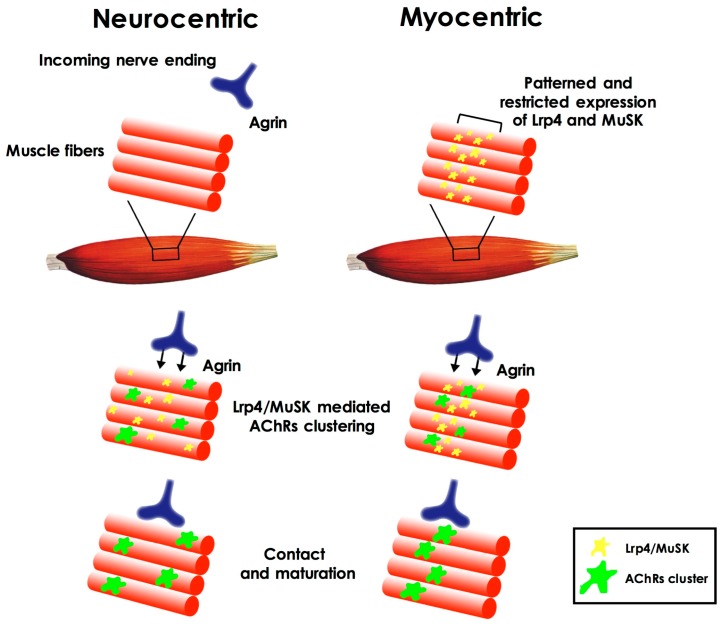
Neurocentric and myocentric hypotheses of neuromuscular synaptogenesis. According to the neurocentric hypothesis, the clustering of the acethylcholine receptors (AChRs) on the muscle fiber plasma membrane is primarily triggered by the release of neural agrin and through activation of the muscle specific kinase (MuSK) and low-density lipoprotein receptor-related protein (Lrp4). According to the myocentric hypothesis, patterned and restricted expression of MuSK and Lrp4 on the fiber plasma membrane defines the area in which neuromuscular synapse will form, before the arrival of the incoming nerve ending (see text).

Which is the first event in the formation of the NMJ has long been considered as a chicken-and-egg problem [4]; which factors and pathways are initially involved in the formation of such synapse during embryonic development (in rodents, between embryonic day 12 and 13.5)? Which of the cell types involved is triggering the event? The first hypotheses about NMJ formation pointed to the prominent role of motor neurons that contacted newly-formed acetylcholine receptor clusters on the muscle plasma membrane (neurocentric hypothesis). According to this view, the clustering of the receptors was induced by the release of agrin by the neuron itself through activation of the muscle specific kinase (MuSK) on the muscle membrane. Subsequently it was shown (both in vitro and in vivo) [5,6,7,8,9] that clusters of acetylcholine receptors (AChRs) could form even in the absence of incoming motor axons and their signaling [6,9], leading to a different hypothesis (known as myocentric) of NMJ formation as a muscle-initiated event. According to this hypothesis muscle derived cues define the area in which NMJ can form on the muscle fiber. These cues are represented by the patterned and restricted expression, on the muscle fiber plasma membrane, of MuSK and Lrp4 (low-density lipoprotein receptor-related protein 4), the agrin co-receptor, and by the agrin-independent activation of MuSK [10] (Figure 1). Importantly, muscle fibers are also responsible for the synthesis and secretion of the extracellular matrix proteins (i.e., laminins) that will compose the synaptic basal lamina, a structure that, in time, has been demonstrated to play a pivotal role in different aspects of NMJ development, such as synaptic initiation, maturation, and stability, but also in maintaining its structural integrity [11].

Both neuro- and myocentric hypothesis, however, did not take into account the role of Schwann cells in NMJ formation. Even if it is now acknowledged that Schwann cells are not necessary for the establishment of the first contact between nerve and muscle [12], recent studies on the development of the NMJ in different animal models have revealed that these cells release factors (i.e., agrin, WNT ligands, neuregulin 1, Glial cell-derived neurotrophic factor (GDNF), and Transforming growth factor (TGF)β) that can influence NMJ assembly and pre- and postsynaptic maturation and, on their turn, TSCs maturation is modulated by pre- and postsynaptically released factors (neuregulin 1, ATP, and laminins) [13].

As it occurs in central synapses, NMJs undergo a long maturation process where the pre- and postsynaptic apparatus are shaped by synaptic activity, whereas the number and size of TSCs change. Finally nerve terminals compete with each other to innervate each muscle fiber and, eventually, in the early postnatal period, this competition will evolve into the elimination of supernumerary inputs. The result of these processes is a scenario in which a single nerve terminal innervates a well-organized postsynaptic specialization in each fiber. Recent studies based on Schwann cell ablation during embryonic and early postnatal life in mice, suggest that during these later phases of NMJ development, TSCs specifically modulate AChRs maturation, and that they are equally important for NMJ stability in adult life [14].

Most of the molecules that play crucial roles in NMJ formation are also essential for its maintenance in adult life where NMJ integrity tightly depends on the presynaptic release of acetylcholine and on the clustering of AChRs on the muscle plasma membrane to trigger muscle action potentials. Loss of function mutations in human genes coding for AChRs subunits, Na_v_1.4 (the sodium channel isoform that is enriched in the postsynaptic cleft’s secondary folds and is essential for the initiation of the muscle action potential), Rapsyn (the AChRs clustering molecule at the postsynaptic membrane), and ColQ (the collagen tail of acetylcholinesterase, the enzyme responsible for the termination of signal transduction in NMJ), all result in the NMJ impairments that are at the basis of most cases of Congenital Myastenic Syndrome (CMS). In addition to these molecular components of the neurotransmission machinery, mutations in genes coding for agrin, Lrp4, MuSK, Dok-7 (a muscle-intrinsic activator of MuSK), and Laminin β2 have also been shown to be associated with CMS [15]. Inducible and/or conditional suppression of the expression of agrin, Lrp4, MuSK, and Laminin β2 in postnatal mice and rats has shown that these molecules are required to maintain adult NMJs [16]. Mature presynaptic elements of the NMJ need the continuous support of the TSCs to be maintained and to finely tune neurotransmission. This has been shown in the adult frog NMJ where, one week after TSCs ablation, a certain degree of retraction of nerve endings, as well as a significant reduction in neurotransmitter release is observed [12].

## 3. The Neuromuscular Junction in Aging

Aging is characterized by a progressive reduction in muscle mass and strength and by a reduced ability of the fibers to regenerate upon injury [16,17]. Similarly, aging is associated with a reduction in the number of motor neurons in the spinal cord [18]. The loss of motor neurons results in the denervation of entire motor units that become re-innervated by the expansion of other pre-existing motor units. The sprouting of new branches from surviving motor axons leads to functional reinnervation of previously-denervated muscle fibers. However, when denervation exceeds the compensatory reinnervation capabilities of aging muscles, denervated muscle fibers are eliminated and the progressive decline in mass and strength become apparent.

While being considered highly stable synapses during a vertebrate life, neuromuscular junctions undergo profound morphological and functional changes in aging. Among the structural alterations of the NMJ that are more frequently observed in aged muscles there is: (1) the increased surface of the postsynaptic area with the degeneration of a subset of junction folds and the fragmentation of the clusters of AChRs together with the insertion or migration of extrasynaptic AChRs in the perijunctional area [16,19]; (2) several AChR clusters are devoid of presynaptic inputs or, more frequently, these are not covering the entire surface of the clusters, leading to a partial focal denervation; (3) some nerve endings are thinner, while others are swollen, compared to those observed in younger muscle NMJs and, occasionally, two nerve endings innervate a single postsynaptic receptor cluster in a configuration the is reminiscent of the poly-neuronal innervation of immature developing NMJ [19]. This failure in the proper re-establishment of the single neuronal innervation might depend on the impairment in the growth of regenerating nerve endings and on the less precise apposition of pre- and postsynaptic specializations that are due to the reduced capability of TSCs to efficiently and precisely re-occupy synaptic sites following denervation. Indeed, in NMJs from aged patients TSC processes were seen to invade the primary synaptic cleft [16].

Reduced levels of the active zone protein Bassoon were detected in presynaptic terminals of aged NMJs, suggesting impairments in active zone formation and synaptic transmission. These data were consistent with the defects in synaptic functions that have been reported in time, namely reduced end-plate potential and stronger synaptic depression after repeated stimulation and reduced frequency of miniature end-plate potentials [20].

To date it is not known whether changes of NMJs in aging muscle are primarily caused by the changes occurring to the motor neuron or to the muscle fiber.

In the past few years, it has been shown that the same factors that are involved in the formation of the NMJ are also needed for its maintenance in adulthood and that altered levels of expression of these factors correlate with the physiological aging of this synapse. Recent data showed that genes coding for different subunits of the AChR (α, δ, γ), as well as MuSK and Lrp4, were significantly up-regulated in aged rats [21,22]. Interestingly these same genes were upregulated upon denervation, thus, these data established a causal link between age-induced sarcopenia and denervation on one hand, while, on the other hand, they suggested that the agrin-MuSK signaling pathway is also involved in age-related changes at the NMJ. Further evidence about the involvement of the Agrin/MuSK/Lrp4 pathway in age-related changes at the NMJ and the reduction in muscle mass come from the observation that, by overexpressing neurotrypsin, the neural serin-protease that is responsible for the inactivating cleavage of agrin, not only are NMJs dismantled in a few days, but a full sarcopenic phenotype is achieved in young adult mice [23].

Cholinergic neurotransmission defects at the NMJ occur with aging but their role in age-related changes at this synapse has long been poorly understood. What is now known is that, by slightly increasing the amount of acetylcholine in the synaptic cleft, adult mice NMJs start to degenerate, suggesting that maintaining normal cholinergic signaling is crucial to slow age-related changes at the NMJ [24].

The extended ACh lifetime in the NMJ cleft led to prolonged currents and potentials. At the same time there is a significant reduction in the amount of available neurotransmitter within the synaptic vesicle due to the impairment in cholinesterase activity [15].

During the period of relative stability of the mature NMJ, all trophic factors and signals deriving from the three partners of the synapse are tightly tuned to maintain an equilibrium that is regulated by synaptic activity and acetylcholine. In aging NMJs this equilibrium is disrupted and, in this context, the circulating levels of neurotrophic and growth factors might be relevant. Indeed, the age-related reduction in the levels of both brain-derived neurotrophic factor (BDNF) and GDNF might play a role in changes of the NMJ in aging [17]. Muscle fibers synthesis and secretion of fibroblast growth factor binding protein 1 (FGFBP1) is restricted to the NMJ area. While the expression of FGFBP1 increases during NMJ development, it decreases in aging fibers and its decrease precedes NMJ alterations in aging. The deletion of FGFBP1 in mice results in defects in NMJ formation and stability [25]. The level of the circulating isoform of insulin-like growth factor 1 (IGF-1) declines with age and this reduction may contribute to NMJ degeneration and muscle fiber denervation. Indeed it was shown that in old mice overexpressing IGF-1 in muscles, the sarcopenic phenotype was abolished and the integrity of NMJ innervation was maintained, and similar results were obtained with the systemic administration of IGF-1 in experimental models of denervation [17]. Another factor that causes a reduction in the level and efficacy of circulating IGF-1 is inflammation and it is known that aging is accompanied by a chronic mild inflammatory state sustained by increased levels of inflammatory cytokines, like interleukin 6 (IL-6), interleukin 1 (IL-1), tumor necrosis factor α (TNFα), and C-reactive protein (CRP) [17].

Mitochondrial dysfunction has long been known to be involved in age-related alteration of the motor system; studies on post-mortem spinal cord samples from the elderly indicated that up to 35% of the motor neurons’ soma contained mitochondria that were lacking in elements of the respiratory chain complexes [26]. However, only recently a systematic study addressed the role of mitochondria in the aging NMJ. Ultrastructural analyses of motor neurons from old rats revealed that in the nerve endings mitochondria presented a number of abnormalities (i.e., swelling and increased size, possibly due to an imbalance in fusion/fission dynamics) that were not observed in the motor neuron soma. Moreover in the cytoplasm of the axon terminals activated caspase 3 and cytochrome C (triggers of the mitochondrial-dependent pathway to apoptosis) were detected, pointing to the nerve ending as the primary site of motor neuron degeneration in aging [27].

The number of mitochondria in skeletal muscle that decrease with age, possibly due to a reduced mitochondrial biogenesis. However, it is still a matter of debate whether this reduction is due to decreased levels of the peroxisome proliferator-activated receptor γ co-activator 1α (PGC-1α) (known as a master regulator of mitochondrial biogenesis) in aging muscles [26].

Several of the events and pathways that are involved in the physiological aging of NMJs and muscles are equally involved in the NMJ defects and skeletal muscle denervation that occur in Amyotrophic Lateral Sclerosis (ALS). Elderly people and ALS patients share common features, starting from reduction in muscle mass and strength, altered metabolism of muscle fibers, which correlates not only with aging, but also with reduced physical exercise, reduced circulating levels of trophic factors, defects in retrograde transport from the nerve ending to the motor neuron soma [28,29,30,31], and reduction in the population of satellite cells around NMJs [32,33].

## 4. Amyotrophic Lateral Sclerosis

Amyotrophic lateral sclerosis (ALS) is an adult-onset, highly debilitating disease that is caused by the progressive degeneration of upper motor neurons in the motor cortex and of lower motor neurons in the brainstem and spinal cord. The progressive failure of the neuromuscular system results in weakness and atrophy of the limb musculature, gradual paralysis, and death from respiratory failure typically within two to three years of symptom onset [34]. Worldwide ALS incidence is about two cases/100,000 per year with a prevalence of five cases/100,000. The majority of ALS cases, about 90%, are sporadic (sALS), while about 10% of ALS cases have a family history of the disease and are classified as familial (fALS) mostly with an autosomal dominant transmission. sALS and fALS share common clinical symptoms [35]. ALS is known to be a complex disease with a multifactorial pathogenesis in which several factors could increase the susceptibility to the disease [36], among those include glutamate-mediated excitotoxicity [37], oxidative stress [38], mitochondrial pool alteration [39] and dysfunction [35], and abnormal protein aggregation [40].

Possibly viral infections [41,42], autoimmune phenomena [43,44], and several other acquired causes linked to environmental conditions [45,46] could also contribute to the disease onset.

In 1993, a landmark discovery of 11 missense mutations in the *SOD1* gene, in 13 fALS families [47], heralded the genetic age for ALS. SOD1 is a ubiquitously expressed metallo-protein with a scavenging effect that functions by detoxifying intracellular superoxide anions. To date, 166 *SOD1* mutations have been reported, accounting for 14–23% familial and 1–7% sporadic ALS cases [48]. It is now known that mutations in a number of different genes cause fALS and contribute to the development of sALS. In addition to *SOD1* mutations, mutations in the *C9orf72* gene, account for 30–40% of fALS in western countries while, worldwide, TARDBP and FUS gene mutations each account for about 5% of all fALS cases. Additionally, the best-known ALS-genes, to date more than 100 gene mutations have been identified, capable of increasing ALS susceptibility or to alter the ALS phenotype in patients. Even if the origin of motor neuron degeneration remains obscure, multiple mechanisms have been proposed to contribute to fALS pathogenesis; among these include excitotoxicity, oxidative stress, defects in protein stability, conformation and aggregation, impairment in cytoskeletal/axonal dynamics, altered RNA metabolism, mitochondrial dysfunction, and altered neuronal excitability [35].

The possibility to study cellular and molecular processes, identify key pathways for intervention, and assess multiple candidate therapies over short periods of time, depends on the availability of animal models for the disease. Forced expression in mice of a human-mutated form of SOD1 (the mutant SOD1 G93A) resulted in the generation of transgenic mice [49] that recapitulates the pathogenic phenotype of ALS patients, namely, an adult onset neurodegenerative disease that is characterized by locomotor impairments, spinal cord and muscular atrophy, motor neuron loss, changes at the NMJ, muscle denervation, and premature death. Rodents [50], zebrafish [51], but also the fruit fly [52,53,54] and the nematode worm [55,56,57], expressing ALS-linked mutated proteins have been used to study the neurobiological basis of ALS. Interestingly, those studies based on animal models have shown that ALS is a non-cell autonomous, multifactorial disease where aberrant behaviors in different cell types, besides motor neurons, are at play and contribute to the onset and progression of the disease. Among them, glial cells, which surround motor neurons and provide nutritional and trophic support [58], TSCs [59], and skeletal muscle fibers [60] could play a crucial role in disease pathogenesis.

## 5. Neuromuscular Junction Degeneration in Amyotrophic Lateral Sclerosis

Despite numerous studies on motor neuron dysfunction in ALS, it is still debated whether motor neuron impairment in ALS has to be considered a dying forward phenomenon, in which primary damages occur in motor neurons in the cortex (i.e., through glutamate excitotoxicity or altered neuronal excitability) and then extend in an anterograde fashion to corticospinal projections [61], or if ALS has to be considered a distal axonopathy in which motor neuron degeneration starts at the nerve endings and progress toward the cell bodies in a dying back manner [62,63]. Given the complexity of ALS pathogenesis it is reasonable to consider that both dying forward and dying back processes can occur independently from each other and, regardless of the progression mode, it is acknowledged that disassembly of the NMJ, leading to skeletal muscle denervation, is a key point in ALS clinical symptoms onset and pathogenesis.

The notion of ALS as a non-cell autonomous disease is based on the observation that, besides motor neurons, other cell types are damaged and display altered behavior both in patients and in vitro/in vivo models of ALS. Moreover, in the past fifteen years, this was shown by expressing ALS-linked mutant proteins in a tissue or in a cell-specific manner [64].

While it is generally accepted that expression of fALS-linked mutated proteins in motor neurons is needed to induce an ALS phenotype in mouse models, it is still debated whether this is a sufficient condition. Initial studies on mice showed that neuron-specific mutant SOD1 expression was not sufficient for the development of the disease [65,66]. This observation was confirmed by the generation of chimeric mice where it was shown that, in the absence of mutant SOD1 expression in non-neuronal cells, mutant SOD1 in neurons was not toxic in itself [67]. On the other hand, a few years later it was reported that limited expression of mutant SOD1 in neurons was sufficient to induce an ALS phenotype in mice [68]. The reasons of this difference might be related to different expression levels of the transgene in the different models. Overall these studies define a scenario in which, in animal models, the expression of mutant SOD1 in neurons is crucial to determine the onset of the disease and the early phases of pathogenesis, whereas expression in non-neuronal cells is relevant to modulate ALS progression.

When analyzing NMJ disassembly in ALS it is necessary to consider the specific role played by the three components of the tripartite synapse in the series of events that culminate in muscle fiber denervation.

Despite the wealth of data describing motor neuron degeneration during ALS pathogenesis, quite a few studies addressed the changes occurring at the motor nerve endings at the NMJ; thus, the series of events that lead to degeneration of the NMJ circuitry is still poorly understood.

Consistent with the dying-back hypothesis in ALS pathogenesis, it was found that distal axonal and NMJ alterations were present in muscles of SOD1 G93A mice before the onset of the clinical symptoms [69]. It was also shown that nerve terminals are particularly sensitive to reactive oxygen species (ROS) accumulation and this suggests that oxidative stress, along with altered mitochondria and increased intracellular Ca^2+^ levels, accelerates the presynaptic decline in NMJ and affects the neurotransmitter release machinery in the presynaptic terminals [70]. Ultrastructural analyses of the presynaptic terminal of NMJs of mutant SOD1 mice at disease onset showed alterations of mitochondria in terms of cristae disorganization, in a subset of nerve endings, with no differences in average area, circularity, and density. In agreement with previous reports, these findings indicated that mitochondrial alterations precede denervation. Synaptic vesicle density in motor axon terminals from mutant SOD1 mice was significantly reduced compared to wild-type ones [39]. The reduction in the size of the synaptic vesicles pool was previously reported based on immunofluorescence experiments in NMJs of mutant SOD1 mice, and it was considered as a consequence of impaired vesicle transport in the axons of fast fatigable motor neurons [71].

Of note, a number of indications about the toxic effects of ALS-linked protein mutations on the development and maintenance of the nerve endings derived from alternative animal models (drosophila and Zebrafish ALS models) where the NMJs are more accessible for analyses in vivo and ex vivo in whole organisms. Indeed, functional and imaging studies have shown that presynaptic motor nerve terminals’ formation and maturation was impaired at the larval muscle NMJ in drosophila models of FUS-mediated ALS [72] and that substantial presynaptic defects were observed at the NMJ in drosophila larvae expressing mutant VAP-B [73].

Expression of mutant SOD1 and reduced expression of TDP-43 in Zebrafish embryos and larvae both resulted in abnormal motor neuron branching and defective formation of presynaptic nerve endings [74,75]. Functional data from TDP-43 downregulated embryos also suggested that reduced levels of this protein cause either increased presynaptic active zones or increased vesicular quantal release.

## 6. The Role of Skeletal Muscle in NMJ Dismantling in Amyotrophic Lateral Sclerosis

Little information is available on the role of muscle integrity in preserving fiber innervation in ALS even if, in mice models of ALS, mutant SOD1 expression in muscle fibers is known to induce a number of toxic effects that mimic those of mutant SOD1 expression in neurons.

As in neurons, the mitochondrial pool is one of the main intracellular targets of SOD1-mediated toxicity in the muscle fiber; thus, structural and functional defects were reported in muscle from sALS patients and transgenic mouse models [60,76], as well as defects in mitochondrial dynamics that can be observed either at presymptomatic stages [77], or can be undetected and become apparent with disease progression [78]. Boosting mitochondrial biogenesis with the overexpression of PGC-1α can restore mitochondrial and muscle functions in SOD1G37R mice without, however, improving mouse survival [79]. Defects of mitochondrial activity in muscles are accompanied by mutant SOD1 cytosolic aggregates formation which, in muscle, are mostly removed by both proteasome- and autophagy-mediated degradation [76] and an increased amount of reactive oxygen species.

It has been shown that muscle-specific mSOD1 expression induces progressive muscle atrophy associated with significant reduction in muscle strength and alterations in the contractile apparatus [80,81]. Similarly, it was shown that defects in muscle metabolism and the reduction in muscle mass can be observed in the SOD1 G93A mice before any evidence of central neuron degeneration [82]. On the contrary, muscle hypertrophy induced by localized expression of insulin-like growth factor-1 (IGF-1) [83] or growth hormone has been shown to exert beneficial effects on fALS mouse survival, especially when associated with moderate physical exercise [80,84].

All of these data suggest that skeletal muscle in ALS can be a primary target of the mutant SOD1 mediated toxicity, however, it is still a matter of debate whether affected muscles play a role in promoting NMJ denervation and motor neuron degeneration. In fact, while muscle-specific expression of mutant SOD1 was initially reported to induce a reduction in muscle mass and strength and mitochondrial dysfunction without effects on motor neuron degeneration [80], later studies indicated instead that overexpression of mutant SOD1 only in muscles was able to faithfully reproduce all major ALS phenotypes, including NMJ alteration and motor neuron degeneration [81].

In support of the idea of a cross talk between motor axon terminals and muscle fibers, it was also shown that overexpression of Vascular endothelial growth factor (VEGF) and GDNF restricted to the skeletal muscle of SOD1 G93A significantly delayed the onset of the disease and increased mouse survival [85,86]. Interestingly VEGF exerted its function in part by counteracting astrogliosis in the CNS and by preventing NMJs’ denervation in the PNS [87].

Recently it has been shown that the reduced levels and secretion of muscle FGFBP1 at the NMJ that is observed in aging and in ALS correlates with defects in NMJ stability. Increasing levels of TGF-β1 in muscle fibers, and specifically at the NMJs, might be at the basis of FGFBP1 decrease [25].

As for NMJ assembly, maintenance and age-related changes, activity might play a role in NMJ survival in ALS pathogenesis. Recent studies highlighted early defects in cholinergic transmission at this synapse in ALS patients and models. Whereas neuromuscular transmission of mutant SOD1 mice was enhanced in the pre-symptomatic phase, it becomes impaired in a subset of NMJs at later stages of the disease [88]. These defects might be related both to pre- or postsynaptic impairments and, indeed, studies were performed to analyze the spatiotemporal expression of both the choline acetyltransferase (ChAT) and vesicular ACh transporter (VAChT) in the motor nerve endings. These two molecules were both downregulated in tissues from patients and mice models strongly suggesting that reduced ACh handling in the presynaptic terminals may contribute to motor neuron distal degeneration [89]. Of note, overexpression of VAChT in SOD1 G93A mice slightly increases the level of ACh in the synaptic cleft of the NMJs, further promoting NMJ degeneration and accelerating disease onset [24]. The integrity of the synaptic basal lamina at the NMJ in ALS has also been investigated, and initial studies on muscle biopsies from ALS patients have indicated a strong reduction in the levels of acetylcholine esterase (AChE). This reduction was accompanied by a marked increase in the plasma level of the circulating enzyme, suggesting a relationship between ALS pathogenesis and protease activity (matrix metalloproteinases) in the NMJ’s synaptic cleft. This evidence was confirmed by observations from patients and models of other neuromuscular diseases. However, muscle fibers are not the only sources of plasma AChE and motor neurons produce and release AChE, whereas TSCs express on their surface butyrylcholinesterase (BChE); thus, it is difficult to define which of the partners of the tripartite synapse is mainly responsible for this alteration [89].

Acetylcholine receptors (AChRs) from ALS patients were mostly characterized by the same pharmacology and the same electrophysiological properties of AChRs from denervated muscles of non-ALS individuals, however, these receptors showed a significant decrease in ACh affinity, compared with denervated non-ALS and a four-fold increase in the expression of the α1 subunit (besides the increase of the γ subunit typical of denervated muscle). Finally, riluzole reduced, in a dose-dependent manner, ACh-evoked currents. These observations support the hypothesis that ALS-mediated denervation of skeletal muscle induces changes at the NMJ that are not shared by denervated muscles after a trauma or because of other neuromuscular pathologies [90,91].

Early studies on muscles from ALS patients showed that the amount and distribution of acetylcholine receptors, as well as the postsynaptic architecture of the NMJ were not significantly altered even in fully-denervated endplates [92,93]. In particular it was demonstrated that, whereas the postsynaptic primary gutter was occasionally flattened (possibly an effect mediated by the reduction in caliber of denervated fibers), secondary folds were always well preserved [94].

Recent findings in SOD1 G93A mice muscles indicated that, before the onset of clinical symptoms, NMJs’ increased branching was accompanied by widespread early axonal and NMJ alterations and immunohistochemical analyses demonstrated that expression levels of the scaffolding proteins nestin, dystrophin, and rapsyn, as well as those of Lrp4, were diminished at different degrees in the gastrocnemius. In the same study it was reported that forelimb muscles showed axonal and NMJ degeneration only later in time, at the post symptomatic stage of the disease and, here, NMJs were characterized by defects in their morphology and reduced complexity [69].

All of these data indicate that muscle fibers have to be considered a primary target of ALS-induced pathogenic events, an important player in ALS initiation and progression, and possibly a target for therapy. However, and even if they do not unambiguously point to muscle fibers as the synaptic component primarily triggering NMJ denervation in ALS, they prove that specific defects in muscle can affect motor nerve ending, thus, contributing to NMJ denervation.

Recent studies on microRNA content in muscle from sporadic and familial ALS patients identified molecular pathways that could affect re-innervation and muscle fiber regeneration which, subsequently, could have an impact in maintaining neuromuscular junction stability in ALS [95].

## 7. Glial Cells, Schwann Cells, and Terminal Schwann Cells in Amyotrophic Lateral Sclerosis Pathogenesis

In the CNS of animal models expressing mutant SOD1, the role of astrocytes has been extensively investigated and it was found that, whereas restricted expression of mutant SOD1 in astrocytes was able to induce astrogliosis, it did not cause motor neuron degeneration [96]. On the other hand, it was shown that these cells played a relevant role in modulating ALS progression, since delayed microglial activation and slowed disease progression in SOD1 mice were observed [97,98] by reducing their level of expression of mutant SOD1. In support of an active role of astrocytes in promoting motor neuron degeneration in ALS, recent data demonstrated that not only astrocytes expressing ALS-associated proteins [99], but also astrocytes from sALS patients, are capable of inducing motor neuron degeneration in vivo [100]. The pathogenic role of mutant SOD1 expression in microglia has been equally investigated and, also in this case, it was show that by downregulating SOD1 levels in microglial cells in vivo increased the mean survival of the mutant mice by slowing disease progression after onset [101,102].

In the peripheral nervous system, the Schwann cells represent the major glial population and can be divided into two classes: (1) myelinating Schwann cells that ensheath lower motor axons regulating their caliber and favoring action potential conduction, and (2) non-myelinating terminal Schwann cells, or perisynaptic Schwann cells, that are needed to support the development, maturation, and maintenance, as well as the regeneration of the NMJs.

The first evidence of the involvement of Schwann cells in ALS came from observations in autoptic samples from ALS patients showing myelin sheet disruption in peripheral nerves, most likely the consequence of axonal degeneration [103]. Later studies in mutant SOD1 mice expressing luciferase under the control of the Glial Fibrillar Acidic Protein (GFAP) promoter showed that, while in the spinal cord, astrocyte activation occurred at presymptomatic stages of the disease, the appearance of the clinical symptoms corresponded with the activation of GFAP expression in myelinating Schwann cells in the peripheral nervous system (PNS) [104]. Transgenic mice overexpressing mutant SOD1 in myelinating Schwann cells were generated to investigate whether Schwann cells play a pathogenic role in the ALS onset and progression by promoting degeneration of NMJs and axons. These animals were indistinguishable from wild type littermates, suggesting that expression of mutant SOD1 in myelinating Schwann cells had no effect on motor neurons and was not linked to ALS pathogenesis [105]. Strikingly, by reducing the expression of a dismutase active mutant SOD1 in myelinating Schwann cells, the progression of the disease is accelerated [106].

Noteworthy, all the data reported are not applicable as such to non-myelinating TSCs, which are known to play pivotal roles in NMJs stability and regeneration and, to date, very little is known about the role of these cells in the pathological changes of the NMJ in ALS.

TSCs play fundamental roles in the physiological re-innervation processes. Upon denervation TSCs switch from a resting (maintenance) to an active (repair) state and this change is triggered by the sudden interruption of the ACh signaling through muscarinic AChRs on the TSCs plasma membrane. TSCs then acquire a macrophage-like behavior and phagocyte axonal and cellular debris; this process is triggered by mitochondrial factors (alarmins) [107] that are released by degenerating axons, and is relevant to promote and facilitate re-innervation. Given the tight relationship between mutant SOD1 toxicity and mitochondrial alterations in neurons [108], these data suggest that debris phagocytosis by TSCs in ALS might be impaired affecting efficient reinnervation processes [109].

Activated TSCs are equally important for the guidance of axonal sprouting to reinnervate the previously-occupied synaptic clefts, to remodel the post-synaptic clusters of AChRs, and to stabilize newly-reinnervated NMJs [110]. These functions, which are mostly mediated by the extension of TSC processes, are phenomena that do not seem to be impaired in ALS mouse models [111]. Interestingly, TSCs switching from maintenance to repair at the NMJ could be dampened instead by an impaired muscarinic activation of the TSCs. In support of this hypothesis is evidence of hyper-muscarinic activity of TSCs in mutant SOD1 mice at disease onset [111]. Thus, persistent muscarinic activation of TSCs might be one of the causes of defects in the NMJ architecture and function in ALS progression.

Deregulated Ca^2+^ homeostasis in glial cells was equally reported as a consequence of over-activation both in astrocytes in the CNS [112] and in TSCs at the NMJs [111]. Given the number and the relevance of the intracellular pathways that are activated/regulated by cytoplasmic calcium concentration it is conceivable that glial over-activation might result in mitochondrial overload, enhanced free radical production, ER, and oxidative stress [59].

Recent data from mutant SOD1 mice have shown that TSCs undergo morphological alteration and reduction in their number before NMJ denervation [113], and many of them further react to denervation by activating their apoptotic pathways instead of promoting reinnervation [114], thus, any motor neuron sprouting from nearby innervated NMJ is unlikely to provide successful compensatory reinnervation in muscles from these mice. On the other hand, reduction in the total number of TSCs might result in alteration of the signaling pathways that are important for NMJ repair and proper function, as, for example, the neuregulin-ErbB pathway. Neuregulin (NRG) and its receptors (ErbBs) are present at adult NMJ with ErbB3 specifically localized at TSCs’ plasma membrane. Evidence from several experiments indicated that the integrity of the NRG-ErbB signaling pathway in TSCs was crucial to maintaining the stability of the NMJ as both its over-activation in ALS and downregulation resulted in synaptic loss [59]. NRG expression is reduced in the spinal cord of ALS patients and mice models and viral mediated delivery on NRG1 in mice could extend mice survival [115]. Disruption of the neuregulin-ErbB4 pathway, induced by the loss of function mutation in the ErbB4 gene, was equally involved in the pathogenesis of ALS [116].

## 8. Conclusions

The assembly, maintenance, and plasticity of neuromuscular junctions are differentially regulated by numerous signaling pathways activated by the cross-talk among the three partners of the NMJ (motor nerve ending, muscle fiber, and terminal Schwann cell) in development (embryonic and early postnatal life), adulthood, and during aging. Interestingly, several of the neuromuscular signaling impairments that lead to age-related changes at the NMJ occur in pathological conditions in which NMJ disassembly and skeletal muscle denervation are key events, like in ALS.

For many years, in the ALS community, researchers tried to identify the cellular component(s) that were primarily responsible for NMJ disassembly in ALS, moving rapidly from the concept of a cell-autonomous to a non-cell-autonomous mechanism, where all three members of this peripheral tripartite synapse play specific roles in ALS onset and progression.

As for NMJ assembly during development, it is now clear that neurocentric or myocentric hypotheses for NMJ dismantling are too tight in their definition and that the trigger for NMJ disassembly in ALS is possibly represented by the cross-talk not only between muscle fiber and motor nerve terminals, but also, as it has clearly emerged in the last few years, in the cross-talk of these two elements with the terminal Schwann cells.
